# Combined therapy with early initiation of infliximab following drainage of perianal fistulising Crohn’s disease: a retrospective cohort study

**DOI:** 10.1186/s12876-021-02078-9

**Published:** 2022-01-10

**Authors:** Ping Zhu, Jin-fang Sun, Yun-fei Gu, Hong-jin Chen, Min-min Xu, You-ran Li, Bo-lin Yang

**Affiliations:** 1 Department of Colorectal Surgery, Affiliated Hospital of Nanjing University of Chinese Medicine, 155 Hanzhong Road, Nanjing, Jiangsu China; 2 Key Laboratory of Environmental Medicine Engineering of Ministry of Education, School of Public Health, Southeast University, Nanjing, Jiangsu China

**Keywords:** Perianal, Fistulising disease, Crohn’s disease, Infliximab, Surgery

## Abstract

**Background:**

Recent studies have confirmed that combined surgery and anti-TNF therapy could improve outcomes in patients with perianal fistulising Crohn’s disease (PFCD). However, the optimal timing for infliximab infusion after surgical intervention is uncertain. We aimed to determine the long-term efficacy of early initiation of infliximab following surgery among PFCD patients.

**Methods:**

We performed a retrospective cohort study of PFCD patients who received combined infliximab and surgical treatment between 2010 and 2018 at a tertiary referral hospital. Patients were grouped according to the time interval between surgery and infliximab infusion, with < 6 weeks into early infliximab induction group and > 6 weeks into delayed infliximab induction group. The primary outcome was to compare surgical re-intervention between early and delayed infliximab induction groups. The secondary outcomes were fistula healing and predictors associated with these outcomes of early infliximab induction approach.

**Results:**

One hundred and seventeen patients were included (73 in early infliximab induction, 44 in delayed infliximab induction). The median interval between surgery and infliximab initiation was 9.0 (IQR 5.5–17.0) days in early infliximab induction group and 188.0 (IQR 102.25–455.75) days in delayed infliximab induction group. After followed-up for a median of 36 months, 61.6% of patients in early infliximab induction group and 65.9% in delayed infliximab induction group attained fistula healing (*p* = 0.643). The cumulative re-intervention rate was 23%, 32%, 34% in early infliximab induction group and 16%, 25%, 25% in delayed infliximab induction group, at 1, 2, and 3 years respectively (*p* = 0.235). Presence of abscess at baseline (HR = 5.283; 95% CI, 1.61–17.335; *p* = 0.006) and infliximab maintenance therapy > 3 infusions (HR = 3.691; 95% CI, 1.233–11.051; *p* = 0.02) were associated with re-intervention in early infliximab induction group. Presence of abscess at baseline also negatively influenced fistula healing (HR = 3.429, 95% CI, 1.216–9.668; *p* = 0.02).

**Conclusion:**

Although no clear benefit was shown compared with delayed infliximab induction group, early initiation of infliximab after surgery could achieve promising results for PFCD patients. Before infliximab infusion, durable drainage is required for patients with concomitant abscess or prolonged infliximab maintenance therapy.

## Background

The estimated incidence of perianal fistulising disease varies from 30 to 50% among patients with Crohn’s disease (CD), which generally indicates aggressive disease patterns and poor prognosis [1–3]. The goal of treatment is to maintain adequate fistula closure and to reduce repeat surgical procedures. Anti-TNF agents (infliximab or adalimumab) are effective in the induction and maintenance of fistula closure and are currently recommended as the first-line medical therapy for perianal fistulising Crohn’s disease (PFCD) [4, 5]. Further data suggested that combining surgery with infliximab could improve fistula closure and prevent fistula recurrence, compared with single treatment alone [6–13].

There is currently no guideline or consensus statement regarding the timing between surgical intervention and commencement of medical therapy [3, 5, 14–17]. The algorithm of early studies included initial drainage with loose seton for two to three months, followed by infliximab infusion or definitive repair of the fistula based on the status of anorectal and intestinal inflammation [18, 19]. However, such treatment modality may bring unnecessary delay in receiving proper medical treatment and increase the risk of poor wound healing. As a tertiary referral center for PFCD, we first brought the concept of early infliximab induction approach into clinical practice in 2010. Infliximab infusion usually started within one week after surgical intervention, resulting in a high fistula closure rate (89.3%) and rapid clinical healing time (average 31 days) [20]. The high healing rate may contribute to the timely initiation of infliximab to control the inflammatory process and promote wound healing. Although no study has formally described this concept by far, most of the recent cohort studies have embraced early infliximab induction approach as the standard management for PFCD patients, with time intervals varying from 24 h to 4 weeks between initial surgery and commencement of medical therapy [9, 21–23]. Further data demonstrated that time interval between surgery and infliximab initiation longer than 6 weeks may negatively impact fistula closure, which underlined the importance of early combined treatment for patients with PFCD [24]. A national survey was designed to collect opinions from consultant gastroenterologists on the management of PFCD. ﻿Most of the respondents described the interval they would normally leave between surgical drainage and commencement of medical therapy less than 6 weeks [25].

The purpose of current study was to compare fistula healing and surgical re-intervention between early and delayed infliximab induction treatment groups in patients with PFCD. We also aimed to identify predictive factors associated with fistula healing and the need for surgical reintervention within the early infliximab induction group.

## Methods

### Patients cohorts and study design

We retrospectively reviewed the medical records of all consecutive patients with perianal Crohn’s disease treated at Affiliated Hospital of Nanjing University of Chinese Medicine, between July 2010 and January 2018. Patients who received combination therapy with surgery and infliximab for PFCD were included. We excluded patients who didn’t complete infliximab induction infusion (5 mg/kg at weeks 0, 2, and 6) and followed up less than 12 months. The diagnosis of PFCD was based on clinical, biological, radiologic, endoscopic, and pathologic evidence.

Two experienced colorectal surgeons (BLY and YFG) were involved in the surgical treatment of all included patients. Perianal surgical procedures included surgical drainage with seton insertion, fistulotomy, rectal advancement flap (RAF), and Ligation of the intersphincteric fistula tract (LIFT). Patients with abscesses underwent surgical drainage first, followed by infliximab infusion. The seton removal was at the discretion of the treating physician following certain principles of our department [20]. Definitive surgical procedures (fistulotomy, RAF, or LIFT procedures) were attempted if no evidence of active proctitis and sepsis exit.

Based on a previous study in which a time interval of 6 weeks between surgery and infliximab initiation was correlated with fistula closure, we defined time interval shorter than 6 weeks as early infliximab induction therapy and longer than 6 weeks as delayed infliximab induction therapy [24].

### Study definition and outcome measures

Fistulas were classified as "simple" or "complex" according to the American Gastroenterology Association (AGA) [26]. Proctitis was defined as ulceration and/or inflammation in the anorectum [5]. Clinical fistula healing was defined as complete closure of the fistula's external opening without discharge or discomfort. The surgical procedure at inclusion was defined as the initial surgery. Additional inpatient surgical procedure for recurrent fistula/abscess after initial surgery was defined as surgical re-intervention (procedures for seton replacement/removal and wound revision were not included). The surgeon who performed the procedure evaluated the patient at every clinic visit or hospital admission for infliximab infusion. A telephone interview was conducted to inquire about the symptoms and maintenance medications at the end of follow-up.

The primary outcome was to compare surgical re-intervention between early and delayed infliximab induction treatment groups. The secondary outcomes were fistula healing and predictors associated with surgical re-intervention and fistula healing of early infliximab induction treatment modality.

### Statistical analysis

Quantitative variables were described as mean ± SD (standard deviation) or median and interquartile range (IQR, P25–P75). Categorical variables were presented as counts and percentages of the cohort. For statistical inference, normally distributed quantitative variables with equal variances were compared using two independent sample *t*-test; otherwise, the Mann–Whitney U test was used. Categorical variables were compared using the chi-square test or Fisher’s exact test. The re-intervention-free survival curves between early infliximab induction and delayed infliximab induction groups were compared using the log-rank test with Kaplan–Meier analysis. Univariate and multivariate logistic regression analyses were performed to identify predictors associated with surgical re-intervention and long-term fistula healing in early infliximab induction cohort. The results were expressed as hazard ratios (HRs) with 95% confidence intervals (CI) and corresponding *p*-value. All statistical analyses were performed using the SPSS 22.0 software (SPSS, Chicago, Illinois, USA), with *p* < 0.05 was considered statistically significant.

## Results

### Patients characteristics and treatment modalities

The full-chart review was performed on 141 patients with perianal Crohn’s disease treated in our center. Among them, 2 patients were excluded for non-fistulising disease, 5 patients for not receiving perianal surgery, 6 patients for incomplete infliximab induction regime, and 11 patients for lack of baseline and/or follow-up data. A total of 117 patients were eventually included, with 73 patients in early infliximab induction group and 44 patients in delayed infliximab induction group. Their baseline characteristics are presented in Table 1.

**Table 1:** Baseline characteristics of early infliximab induction and delayed infliximab induction groups at infliximab initiation

Characteristics	Early infliximab induction (n = 73)	Delayed infliximab induction (n = 44)	*p *value
Sex (male), n (%)	56 (76.7)	28 (63.6)	0.128
Age at inclusion, years (IQR)	25.0 (20.0–29.0)	24.0(21.0–31.75)	0.547
BMI, kg/m^2^ (mean ± SD)	18.98 ± 2.77	19.12 ± 2.91	0.810
Mean disease duration, months (IQR)			
Luminal disease	9.0 (1.0–39.0)	4.5 (1.0–24.0)	0.294
Perianal disease	12.0 (3.0–35.0)	19.0 (5.25–30.5)	0.354
Smoking status, n (%)			0.511
Non-smokers	69 (94.5)	39 (88.6)	
Ex-smokers	3 (4.1)	4 (9.1)	
Active smokers	1 (1.4)	1 (2.3)	
Initial presentation of CD, n (%)			0.029
Perianal	40 (54.8)	33 (75.0)	
Luminal	33 (45.2)	11 (25.0)	
Types of fistula, n (%)			0.614
Anorectal	68 (93.2)	39 (88.6)	
Invade to adjacent organ^a^	5 (6.8)	5 (11.4)	
Complex fistulas (AGA), n (%)	66 (90.4)	40 (90.9)	1.000
Proctitis, n (%)	18 (24.7)	12 (27.3)	0.828
Anorectal stricture, n (%)	27 (37.0)	9 (20.5)	0.067
Presence of abscess at baseline, n (%)	42(57.5)	20(45.5)	0.205
Previous perianal surgery, n (%)	39 (53.4)	22 (50.0)	0.849
Disease behavior, n (%)			1.000
B1-inflammation	47 (64.4)	28 (63.6)	
B2-stricturing	25 (34.2)	16 (36.4)	
B3-penetrating	1 (1.4)	0 (0)	
Disease location, n (%)			0.447
L1-ileal	16 (21.9)	10 (22.7)	
L2-colonic	10 (13.7)	3 (6.8)	
L3-ileocolonic	40 (54.8)	29 (65.9)	
L4-upper gastrointestinal	7 (9.6)	2 (4.6)	
Previous medical treatment, n (%)			0.753
None	31 (42.5)	22 (50.0)	
Infliximab	2 (2.7)	2 (4.5)	
Immunomodulators	10 (13.7)	6 (13.6)	
5-ASA	30 (41.1)	14 (31.8)	

*IQR* interquartile range, *SD* standard deviation, *BMI* body mass index, *CD* Crohn’s disease, *AGA* American Gastroenterology Association, *5-ASA* 5-aminosalicylic acid

^a^Vagina or urethra

There were no differences in patient demographics, previous surgical and medical management, fistula complexity, proctitis, and luminal phenotype between two groups. The median time interval between initial surgical procedures and first infliximab infusion was 9.0 (IQR 5.5–17.0) days in early infliximab induction group and 188.0 (IQR, 102.25–455.75) days in delayed infliximab induction group. The proportion of anorectal stricture tended to be higher in early infliximab induction group than in the delayed infliximab induction group (37% vs. 20.5%, *p* = 0.067). In early infliximab induction group, seton placement were more often used (95.9% vs. 75%, *p* = 0.001), while fistulotomy were less often utilized (2.7% vs. 18.2%, *p* = 0.011) (Table 2) compared to delayed infliximab induction group.

**Table 2:** Comparison of outcomes between early infliximab induction and delayed infliximab induction groups

Characteristics	Early infliximab induction (n = 73)	Delayed infliximab induction (n = 44)	*p* value
Duration of follow-up, months (IQR)	32.0 (21.0–60.5)	37.5 (33.5–57.25)	0.021
Initial surgery, n (%)			0.001
Seton drainage	70 (95.9)	33 (75.0)	0.001
Fistulotomy	2 (2.7)	8 (18.2)	0.011
RAF	0 (0)	2 (4.5)	0.296
LIFT	1 (1.4)	1 (2.3)	1.000
Medical treatment at last follow-up, n (%)			0.761
Infliximab	10 (13.7)	3 (6.8)	0.524
Immunomodulators	33 (45.2)	21 (47.7)	0.979
5-ASA	4 (5.5)	3 (6.8)	0.934
None	26 (35.6)	17 (38.6)	0.743
Re-intervention, n (%)	25 (34.2)	11 (25.0)	0.294
Fistula healing, n (%)	45 (61.6)	29 (65.9)	0.643
Re-intervention-free healing rate, n (%)	33 (45.2)	24 (54.6)	0.328

*IQR* interquartile range, *RAF* rectal advancement flap, *LIFT* ligation of the intersphincteric fistula tract, *5-ASA* 5-aminosalicylic acid

**Fig. 1:**
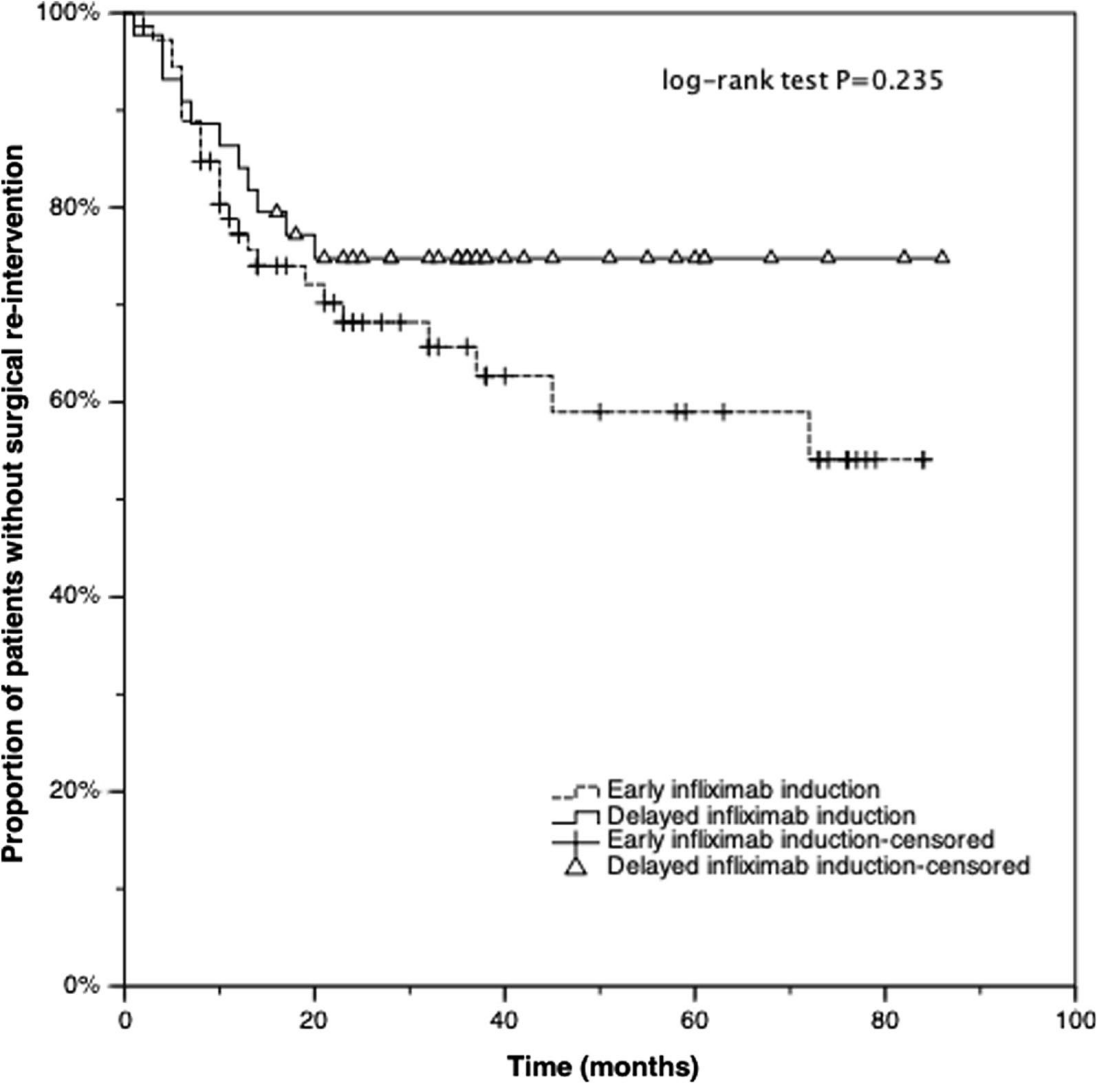
Kaplan–Meier curves comparing the cumulative probability of remaining on re-intervention free status after initial surgery between early infliximab induction and delayed infliximab induction groups

After a median follow-up of 36.0 (IQR 23.5–58.5) months after infliximab initiation, a total of 36 (30.8%) patients had undergone at least one additional perianal procedure after initial surgery. No significant difference in surgical re-intervention rate was found between two groups (34.2% vs. 25%, *p* = 0.294). The cumulative proportion of patients who remained free of re-intervention after initial surgery was demonstrated with Kaplan–Meier survival curves, and there was no significant difference between two groups by using the log-rank test (*p* = 0.235) (Fig. 1). The cumulative probability of receiving surgical re-intervention was 23%, 32%, 34% in early infliximab induction group and 16%, 25%, 25% in delayed infliximab induction group, at 1, 2, and 3 years respectively.

Seventy-four (63.2%) patients have maintained fistula healing at the end of follow-up. Among them, 57 (48.7%) patients have maintained fistula healing without any re-intervention after initial surgeries (re-intervention-free fistula healing). No differences in total fistula healing (61.6% in early infliximab induction group *vs* 65.9% in delayed infliximab induction group, *p* = 0.643) and re-intervention-free fistula healing (45.2% in early infliximab induction group vs. 54.6% in delayed infliximab induction group, *p* = 0.328) were found between different treatment groups (Table 2). At last follow-up, only 11% of patients were still maintaining with infliximab. The proportion of patients with ongoing infliximab treatment was similar between two groups (13.7% in early infliximab induction group and 6.8% in delayed infliximab induction group, *p* = 0.524). Forty-six percent of patients had converted to immunomodulators, including azathioprine (35.9%) and thalidomide (9%). No difference was detected in patients maintained with immunomodulators between two groups (Table 2). Meanwhile, 36.8% of patients had stopped medical therapy after thorough evaluation and discussion between patients and physicians. We did not observe difference in patients who had stopped medication (35.6% in early infliximab induction group and 38.6% in delayed infliximab induction group, *p* = 0.743).

### Risk factors associated with surgical re-intervention and fistula healing of early infliximab induction treatment modality

We then explored the risk factors associated with surgical re-intervention in early infliximab induction group. On univariate analysis, presence of abscess at baseline (HR = 4.727; 95% CI 1.523–14.672; *p* = 0.007) and infliximab maintenance therapy more than 3 infusions (HR = 3.243; 95% CI 1.169–8.996; *p* = 0.024) were related to the surgical re-intervention. On multivariate analysis, presence of abscess at baseline resulted in a fivefold increased risk of re-intervention (HR = 5.283; 95% CI 1.61–17.335; *p* = 0.006), and infliximab maintenance therapy more than 3 infusions increased over threefold risk of re-intervention (HR = 3.691; 95% CI 1.233–11.051; *p* = 0. 02) during the follow-up period (Table 3). When concerning clinical fistula healing in early infliximab induction group, presence of abscess at baseline was negatively associated with fistula healing in univariate and multivariate analysis (HR = 3.429, 95% CI 1.216–9.668; *p* = 0.02) (Table 4).

**Table 3:** Univariate and multivariate analyses on predictors of surgical re-intervention in early infliximab induction group (n = 73)

Variable	Univariate analysis		Multivariate analysis	
	HR (95%CI)	*p* value	HR (95%CI)	*p* value
Sex (male)	0.941 (0.302–2.938)	0.917		
Age at inclusion	0.983 (0.92–1.05)	0.614		
Complex fistula	3.429 (0.389–30.197)	0.267		
Proctitis	0.698 (0.260–1.872)	0.475		
Anorectal stricture	0.938 (0.343–2.559)	0.9		
Presence of abscess at baseline	4.727 (1.523–14.672)	0.007	5.283 (1.61–17.335)	0.006
Previous perianal surgery	0.917 (0.348–2.414)	0.86		
Disease behavior				
B1-inflammation				
B2/B3-stricturing/penetrating	0.594 (0.208–1.692)	0.329		
Disease location		0.329		
L1-ileal				
L2-colonic	2.2 (0.431–11.219)	0.343		
L3-ileocolonic	0.834 (0.236–2.955)	0.779		
L4-upper gastrointestinal	2.933 (0.469–18.333)	0.25		
Maintenance with infliximab				
≤ 3 infusions				
> 3 infusions	3.243 (1.169–8.996)	0.024	3.691 (1.233–11.051)	0.020

*HR* hazard ratio, *CI* confidence interval

**Table 4:** Univariate and multivariate analyses on predictors of long-term fistula healing in early infliximab induction group (n = 73)

Variable	Univariate analysis		Multivariate analysis	
	HR (95%CI)	*p* value	HR (95%CI)	*p* value
Sex (male)	0.456 (0.152–1.373)	0.163		
Age at inclusion	1.042 (0.977–1.111)	0.213		
Complex fistula	1.625 (0.293–9.007)	0.578		
Proctitis	1.2 (0.452–3.186)	0.714		
Anorectal stricture	2.462 (0.922–6.572)	0.072		
Presence of abscess at baseline	3.429 (1.216–9.668)	0.02	3.429 (1.216–9.668)	0.02
Previous perianal surgery	0.8 (0.311–2.06)	0.644		
Disease behavior				
B1-inflammation				
B2/B3-stricturing/penetrating	2.133 (0.798–5.704)	0.131		
Disease location		0.752		
L1-ileal				
L2-colonic	1.111 (0.22–5.616)	0.899		
L3-ileocolonic	0.897 (0.27–2.988)	0.86		
L4-upper gastrointestinal	2.222 (0.365–13.538)	0.386		
Maintenance with infliximab				
≤ 3 infusions				
> 3 infusions	1.319 (0.512–3.396)	0.566		
Need re-intervention	2.383 (0.882–6.440)	0.087		

*HR* hazard ratio, *CI* confidence interval

## Discussion

Patients with PFCD represent a more aggressive and disabling disease course [2]. To reduce the need for multiple operations and associated comorbidities, a combination of surgery with anti-TNF agents targeted at optimization of perianal and luminal diseases simultaneously has been suggested as the preferred treatment modality [3, 5, 16, 17]. An important factor that should be taken into consideration when adopting combination therapy is the time interval between surgical and medical treatments. In the current study, we directly compared early infliximab induction approach with delayed infliximab induction approach. At a median follow-up of 3 years, the fistula healing rate in both groups was beyond 60% (61.6% in early infliximab induction vs. 65.9% in delayed infliximab induction). Our finding is similar to a retrospective study also using early infliximab induction therapy, with two to four weeks between fistula surgery and infliximab induction therapy. Fifty-nine percent of patients with PFCD completely healed after a combination of operative treatment and infliximab [23]. Some recent studies have highlighted the benefit of early introduction of the biological agent after surgical drainage of sepsis [9, 20–24]. The essential reason to support the concept of early infliximab induction therapy is the rapid therapeutic response of infliximab for both luminal and fistulous disease [18, 27]. In an observational study of 129 patients, clinical response and remission for the fistulous disease occurred at a median of 9 days (ranging from 5 to 47 days) and 10 days (ranging from 6 to 54 days) respectively [28]. In ACCENT I trial, 58% of patients responded to a single infusion of infliximab within 2 weeks [29]. No difference could be demonstrated regarding fistula healing between two combination approaches. It needs to be emphasized that significantly more patients in delayed infliximab induction group had fistulotomy as their initial surgery than in early infliximab induction group (18.2% vs. 2.7%). Definitive surgeries (such as fistulotomy, RAF, or LIFT) are justified by their effectiveness of increasing fistula healing and decreasing repeat surgery for PFCD, compared with seton drainage along [18, 21, 23]. However, Definitive surgery could only be attempted in highly selected simple fistula without proctitis and abscess. For complex PFCD, placement of a non-cutting seton is still the treatment of choice. Almost all patients (95%) in early infliximab induction group received seton placement as the initial surgery, still achieved a 62% fistula healing rate. The promising result may attribute to the multimodality approach we applied in daily practice, which included MRI-guided drainage of all sepsis and early initiation of anti‐TNF agent, followed by early removal of seton. Our findings indicated that timing instead of type of intervention may play an important role in managing PFCD.

Heterogeneity in outcome definition hampers effective data analysis and comparison between different studies about PFCD treatments. In solutions try to address this issue, we used the need for fistula‐related re-intervention as the primary endpoint. Since repeat surgery may act as a surrogate marker for fistula relapse and avoiding this event is the essential goal for PFCD management [30–32]. In the current study, 30% of patients required at least one repeat surgery through the follow-up period. There was a trend toward a higher re-intervention rate in early infliximab induction group (34%) as compared with delayed infliximab induction group (25%). This may be due to the higher proportion of anorectal stricture and abscess in early infliximab induction group, which increased the complexity of management. However, no significant difference in total and cumulative re-intervention was found between the two groups. Our finding is similar as recently reported by a multicenter study from Europe, 32% of PFCD patients who received multimodal treatment required repeating perianal fistula‐related surgery [32].

Identifying the predictors affecting long-term outcomes of early infliximab induction therapy could provide useful point for directing personalized therapy. However, the relevant study focus on this field is scarce [21, 24, 33]. Previous studies have identified multimodality treatment, seton removal, therapy with biological agents, and complete fistula response was associated with reduced surgical intervention [32, 34]. In our study, the presence of abscess at baseline increased the risk of surgical re-intervention and adversely affected long-term fistula healing in early infliximab induction group. Concerns have been raised that use of infliximab might increase perianal abscess formation or fistula recurrence because of the rapid closure of the external fistula opening and persistent inflammation within the residual tract [35, 36]. Literature remain controversial regarding the impact of infliximab on perianal infectious complications. Two population-based studies have observed the rising trend of abscess drainage since the introduction of infliximab [37, 38]. On the contrary, data from the ACCENT II study failed to demonstrate the association between abscess development and infliximab therapy [39]. Our findings indicate that for patients associated with abscess at the time of surgery, starting infliximab infusion too early may increase the risk of repeat surgery and compromise fistula healing. In a small case series of early infliximab induction therapy, 12 of 22 complex perianal fistula had abscess that required drainage. Long-term clinical fistula healing was only achieved in 18% of patients [9]. One could speculate that the concomitant abscess cavity warrants a longer time of drainage to allow the inflammatory process to settle down before infliximab therapy is scheduled.

Infliximab maintenance therapy more than 3 infusions was another risk factor for surgical re-intervention in early infliximab induction group. Unlike previous studies, which showed that maintenance therapy with infliximab could reduce the risk of surgery and improve fistula closure in patients with PFCD [4, 24]. This difference could be due to the potential increased risk of abscess formation with infliximab maintenance therapy, which also involves repeat surgical drainage. In our study, only 13% of patients in early infliximab induction group had ongoing infliximab therapy at the last follow-up, while almost half of the patients had switched to immunomodulators. Several cohort studies have demonstrated promising fistula healing in patients who only received induction or short-term infliximab maintenance therapy, followed by immunomodulators [9, 18, 23]. Therefore, we hypothesized that early use of biologics for a limited duration to obtain a quick response, followed by cheaper immunosuppressant agents, could be considered as a cost-effective alternative for patients with PFCD.

Our study has several limitations: (1) a higher proportion of our cases received early infliximab induction approach leading to a significantly smaller delayed infliximab induction group and making it difficult to obtain a statistical difference between some results. (2) Due to the retrospective feature, objective fistula severity measurements such as MRI score or PDAI score were not routinely collected during our study. Although rectal MRI was performed in all included patients before treatment, a great percent of patients didn’t receive MRI re-examination during follow-up. (3) All cases were collected from a single tertiary academic center, leading to the inclusion of a high number of patients with complex fistulas, and whether these data apply to patients with simple PFCD is uncertain. (4) The time interval of combination therapy was wide between different patients, especially in the delayed infliximab induction group; however, this may reflect the complexity of decision-making in PFCD management. (5) We only used 'external opening closure' as the marker of fistula healing in the current study, which is not sufficient in outcome measure for PFCD as highlighted by a recent study [40]. Compressive outcome measures including a patient-reported outcome measure (PROM) may complement the objective clinical evaluation of PFCD patients. (6) We didn’t include the data about the use of antibiotics in the current study. We believe that antibiotics are not necessarily required when surgical drainage is sufficient. Therefore, we do not routinely prescribe antibiotics for PFCD patients after surgical intervention.

The strengths of this study include (1) the relatively large sample size in a single institution with two experienced surgeons performing all of the surgeries. (2) We measured perianal surgical re-intervention and long-term clinical fistula healing as endpoint definitions, which are clinically relevant parameters in reflecting real-world practice. (3) We only included patients who have finished infliximab induction therapy and have been followed up over one year, which was robust enough to provide reliable long-term outcomes.

## Conclusions

In conclusion, early initiation of infliximab therapy after surgery could result in long-term fistula healing in a significant proportion of PFCD patients with an acceptable surgical re-intervention rate. For patients with concomitant perianal abscess or requiring prolonged infliximab maintenance therapy, a longer time interval is warranted to establish durable drainage before infliximab therapy is initiated. For patients with PFCD who receive early infliximab induction therapy, maintenance with immunomodulators such as azathioprine could be a reasonable alternative. The optimal timing to initiate medical treatment needs to be determined in future studies.

## Data Availability

The datasets used and/or analysed during the current study are available from the corresponding author on reasonable request.

## Abbreviations

CD: Crohn’s disease
PFCD: Perianal fistulising Crohn’s disease
RAF: Rectal advancement flap
LIFT: Ligation of the intersphincteric fistula tract
AGA: American Gastroenterology Association
SD: Standard deviation
IQR: Interquartile range
HRs: Hazard ratios
CI: Confidence intervals
BMI: Body mass index
5-ASA: 5-Aminosalicylic acid
